# Unmet medical needs definition and incentives: stakeholders perspectives on the reform of the EU pharmaceutical legislation

**DOI:** 10.3389/fmed.2024.1506243

**Published:** 2025-01-07

**Authors:** Io Wens, Zilke Claessens, Alice Vanneste, Liese Barbier, Rosanne Janssens, Isabelle Huys

**Affiliations:** Department of Pharmaceutical and Pharmacological Sciences, KU Leuven, Leuven, Belgium

**Keywords:** unmet medical need, pharmaceutical legislation, healthcare policy, medicines development, patient need, innovation, sustainability, incentives

## Abstract

**Introduction:**

The 2020 pharmaceutical strategy for Europe stressed that rethinking regulatory policies to foster innovation in disease areas with unmet medical needs (UMN) is one of the European Commission’s (EC) priority areas. To understand stakeholders’ views regarding appropriate UMN criteria and incentives, the EC developed a survey and launched it for public consultation between September and December 2021. This study aims to assess stakeholders’ views on the policy revisions proposed by the EC, particularly those regarding the definition of UMN, its criteria and incentives and evaluate how stakeholders’ views are reflected in the proposed reform of the EU pharmaceutical legislation of 2023.

**Methods:**

The public consultation survey comprised 14 questions including multiple-choice and open answer questions about the reform of the pharmaceutical legislation. A mixed-method analysis was conducted on publicly available data of stakeholders’ responses, including descriptive and quantitative statistics for multiple-choice questions and a qualitative thematic framework analysis for open answer questions. A subgroup analysis was performed to assess differences and similarities in stakeholders’ views, and results were compared with the proposed reform of the EU pharmaceutical legislation.

**Results:**

A total of 478 participants completed the survey consisting of 36% industry, 19% end-users, 17% healthcare providers, 7.5% researchers and 7.5% public bodies. All stakeholder groups favored including “absence of satisfactory authorized treatment” and “disease seriousness” as defining criteria for UMN. However, stakeholders disagreed on including the criterion “lack of access for patients,” with public bodies and industry being less in favour. Industry favored maintaining or having additional incentives like transferable exclusivity vouchers on top of current intellectual property rights to foster innovation. In contrast, other stakeholders supported alternative proposals, namely enhancing the use of scientific advice and implementing expediting measures for regulatory evaluation of medicines targeting UMN.

**Conclusion:**

Stakeholders agreed on including availability of alternatives and disease seriousness in the UMN definition but highlighted its ambiguity. Industry participants supported additional incentives like transferable exclusivity vouchers, whereas others preferred scientific and regulatory support. These findings underscore the need for further discussion on UMN criteria and incentives to stimulate innovation while ensuring patient-centric outcomes and equitable access to medicines across Europe.

## 1 Introduction

**Figure 1:**
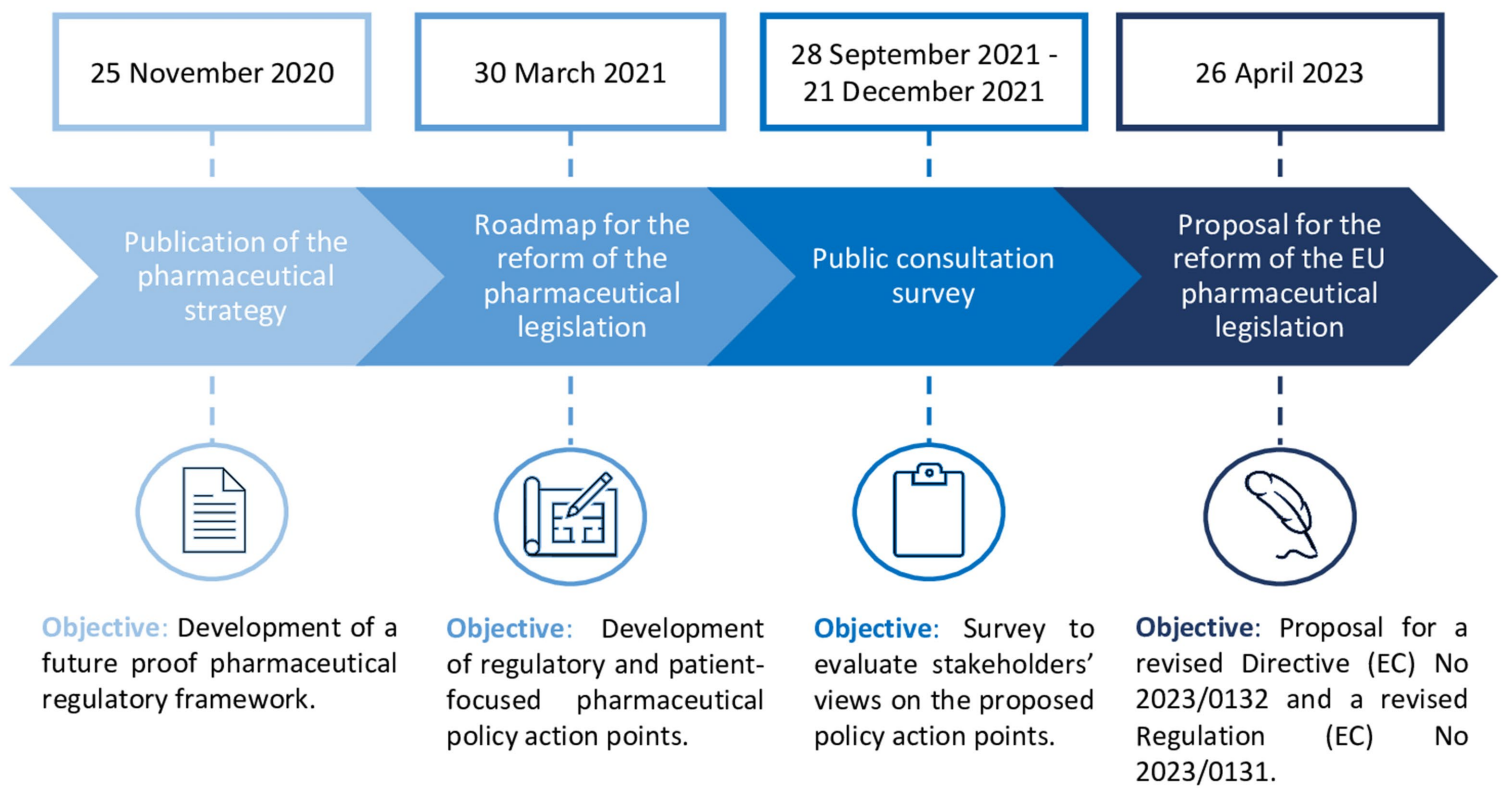
Timeline of the European Commission’s proposal for the reform of the EU pharmaceutical legislation (4).

In response to the COVID-19 pandemic, Europe has been re-evaluating its regulatory and health policy framework, resulting in proposals for significant legislative changes, especially in pharmaceutical development. This began with the publication of the Pharmaceutical Strategy of 2020 for Europe, describing general policy initiatives for developing a patient-centered, future proof and crisis-resistant pharmaceutical regulatory framework (1). The aims of the pharmaceutical strategy were (i) ensuring timely and equitable access to safe medicines across the EU, (ii) enhancing supply security regardless of geographical location, (iii) fostering innovation in medicine research and production, (iv) promoting environmental sustainability, and (v) addressing antimicrobial resistance (AMR) and pharmaceutical pollution (1). To achieve these objectives, the European Commission (EC) published its roadmap for the reform of the existing EU pharmaceutical legislation (Regulation EC726/2004, Directive EC83/2001), proposing concrete policy priority areas for legislative change (Figure 1) (2). Subsequently, the EC developed a survey which was made available for public consultation between September and December 2021, containing concrete policy proposals related to the reform of the EU pharmaceutical legislation. This public consultation aimed to collect views of stakeholders and members of the general public on the pharmaceutical policy measures proposed by the EC. On the 26th of April 2023, the EC published its proposal for the reform of the EU pharmaceutical legislation (2).

In both the Pharmaceutical Strategy of 2020 for Europe and the proposal for the reform of the pharmaceutical legislation, there is a notable increased emphasis on strategies to steer research and development (R&D) to address unmet medical needs (UMN) (1, 2). The EC highlights the importance of addressing UMN, as many patients suffering from serious diseases still lack appropriate treatments and current investments in developing medicines do not always prioritize the greatest UMN. Moreover, the EC’s proposal for the reform of the EU pharmaceutical legislation aims to shift innovation from a supply-driven model to a more needs-driven approach, and contribute to better serving patients and health systems (2).

Currently, the EC defines the concept of UMN as *“a condition for which there exists no satisfactory method of diagnosis, prevention or treatment authorized in the Community or, even if such a method exists, in relation to which the medicinal product concerned will be of major therapeutic advantage to those affected.”* (3). This concept has been officially applied as an eligibility criterion for innovative medicines to facilitate marketing authorization under the form of conditional marketing authorization^1^ (3, 4). Additionally, it has been informally applied in various regulatory practices such as accelerated assessments, priority medicines (PRIME) scheme, authorizations under exceptional circumstances, and scientific advice procedures. Thus, already today the UMN concept enables, to a certain extent, regulatory flexibilities to support development and evaluation of medicines targeting UMN. However, in 2019, Vreman et al. reported differing understanding between stakeholders on the UMN concept, its scope and practical application in regulatory frameworks (5).

The EC’s proposal for the reform of the EU pharmaceutical legislation includes a new definition for UMN [Proposal for a Directive (EC) No 2023/0132, Art. 83(1)] and, within the context of rare diseases, an additional definition for high UMN [Proposal for a Regulation (EC) No 2023/0131, Art. 70(1)] (6, 7). Nevertheless, considerable reaction and commentary has emerged on the proposed legislation, particularly concerning the UMN definition and its connection to anticipated incentives for medicines aimed at addressing these needs (8). Whilst agreement exists that targeted incentive measures are key to fostering innovation in pharmaceutical development, it remains questionable which type of incentive measures are most appropriate to steer innovation in disease areas with UMN (9, 10). The diverging interpretation of the UMN concept results in a lack of systematic interpretation and application of the UMN definition and its associated incentives in practice (5).

This study aimed to assess the views of stakeholders (including industry, public bodies, patients, healthcare providers and researchers) and the general public on the EC’s policy proposals outlined in the Pharmaceutical Strategy of 2020 for Europe regarding (i) general perceptions on the UMN definition, (ii) criteria to characterize UMNs, and (iii) incentive measures to support innovation in UMN areas. Additionally, the study seeks to perform an inter-stakeholder comparison to understand differing perspectives and assess how these are reflected within the proposed reform of the EU legislation. Finally, this study aims to formulate actionable recommendations based on these insights.

## 2 Materials and methods

This study consisted of (i) a quantitative and qualitative analysis of stakeholders’ responses on the EC’s public consultation survey in preparation of the reform of the pharmaceutical legislation and (ii) a comparison of stakeholders input with the final content included in the proposed reform of the EU pharmaceutical legislation published in April 2023. It is important to note that this research is a secondary analysis of publicly available data, not primary research. While the EC has published a summary report of this data, this study provides an additional independent academic examination of the empiric data focusing on the UMN definition and related incentives to steer R&D (11). This study supplements the EC summary report with additional quantitative assessments and in-depth inter-stakeholder comparisons of both quantitative and qualitative data.

### 2.1 Public consultation survey analysis

The public consultation survey consisted of 14 questions including 10 multiple-choice questions and 4 open-ended questions. Each multiple-choice question contained several multiple-choice sub-questions as well as an open-ended answer field in which respondents could further clarify their choice. For the scope of this research, survey questions 1, 3, 4 and question 14 were analyzed as they primarily focused on proposed policy measures for (i) defining the concept of UMN and (ii) potential regulatory incentive measures for driving pharmaceutical development. More specifically, questions 3 and 4 were multiple-choice questions related to the UMN definition and incentives to drive R&D, with both questions also containing an open answer box. Questions 1 and 14 were open-ended questions which were also screened for input relating to the research topic. The exact survey questions can be found in Supplementary Table S1.

The data extraction table including all stakeholders’ responses to the public consultation was consulted via the website of the EC and used for secondary analysis (1). Respondents were categorized into five overarching stakeholder clusters including (1) public body, (2) industry, (3) researchers, (4) end-users, (5) healthcare providers (HCPs). This cluster classification was performed based on which stakeholder subtype respondents mostly identified itself with. All respondents who identified as “other” in the public consultation survey were clustered separately as “other” and their responses were excluded from the analysis. The cluster classification maintained in this analysis slightly differs from the EC’s summary report as the EC screened respondents who identified as “other” and partially re-allocated them to another stakeholder group, causing slight differences in the included number of respondents per cluster (11).

#### 2.1.1 Quantitative analysis

The answer options from the multiple-choice questions included in this analysis (*n* = 2) were scored from 0 to 5 using the VLOOKUP formula in Microsoft Excel (Table 1).

**Table 1:** Scoring of multiple-choice answer options using the VLOOKUP formula.

Score	Multiple-choice answer option
0	Do not know
1	Not important
2	Slightly important
3	Fairly important
4	Important
5	Very important

For each stakeholder cluster, the individual scores from respondents were summed to calculate the average score for each stakeholder cluster per question. The multiple-choice sub-questions that were answered with “do not know” (i.e., score 0), were excluded from this calculation as they would negatively impact the calculated average score. Additionally, the overall average of these stakeholder group averages was calculated. Subsequently, heatmaps were developed in Microsoft Excel to visualize each stakeholder cluster’s level of satisfaction with the proposed policy measure. The conditional formatting tool in Excel was used to automatically color (i.e., green, orange, yellow and red) each average value in the heatmap relative to another.

#### 2.1.2 Qualitative analysis

An extraction table was made in Microsoft Excel including all responses on the open-ended questions (*n* = 586) as well as the open-answer text fields (*n* = 500) of the multiple-choice questions per stakeholder cluster. Subsequently, a thematic framework analysis was conducted, and inductive coding was performed to categorize and classify stakeholders’ responses under specific topics (2). A framework matrix was developed and the answers for each question were summarized per stakeholder cluster to perform an inter-group comparison of stakeholder responses.

### 2.2 Comparative analysis with the proposed revised legislation

A systematic comparison was conducted between analyzed average quantitative results and qualitative stakeholder suggestions and the formulation of the UMN definition, its criteria and proposed incentives included in the proposal for the reform of the EU pharmaceutical legislation. First, specific stakeholder recommendations relating to revisions of the EU pharmaceutical legislation were identified. Secondly, a side-by-side comparison of these stakeholder recommendations with the legislative proposal was performed. To do so, the proposed Regulation [Proposal for a Regulation (EC) No 2023/0131] and Directive [Proposal for a Directive (EC) No 2023/0132] included in the reform of the EU pharmaceutical legislation were reviewed to identify legal changes compared to the existing pharmaceutical legislation. The comparison evaluated the degree of alignment between stakeholder recommendations and the proposed policy changes, noting where stakeholder input was directly incorporated, where modifications were made and potentially suggestions were indirectly or implicitly included, and where suggestions were excluded.

## 3 Results

**Figure 2:**
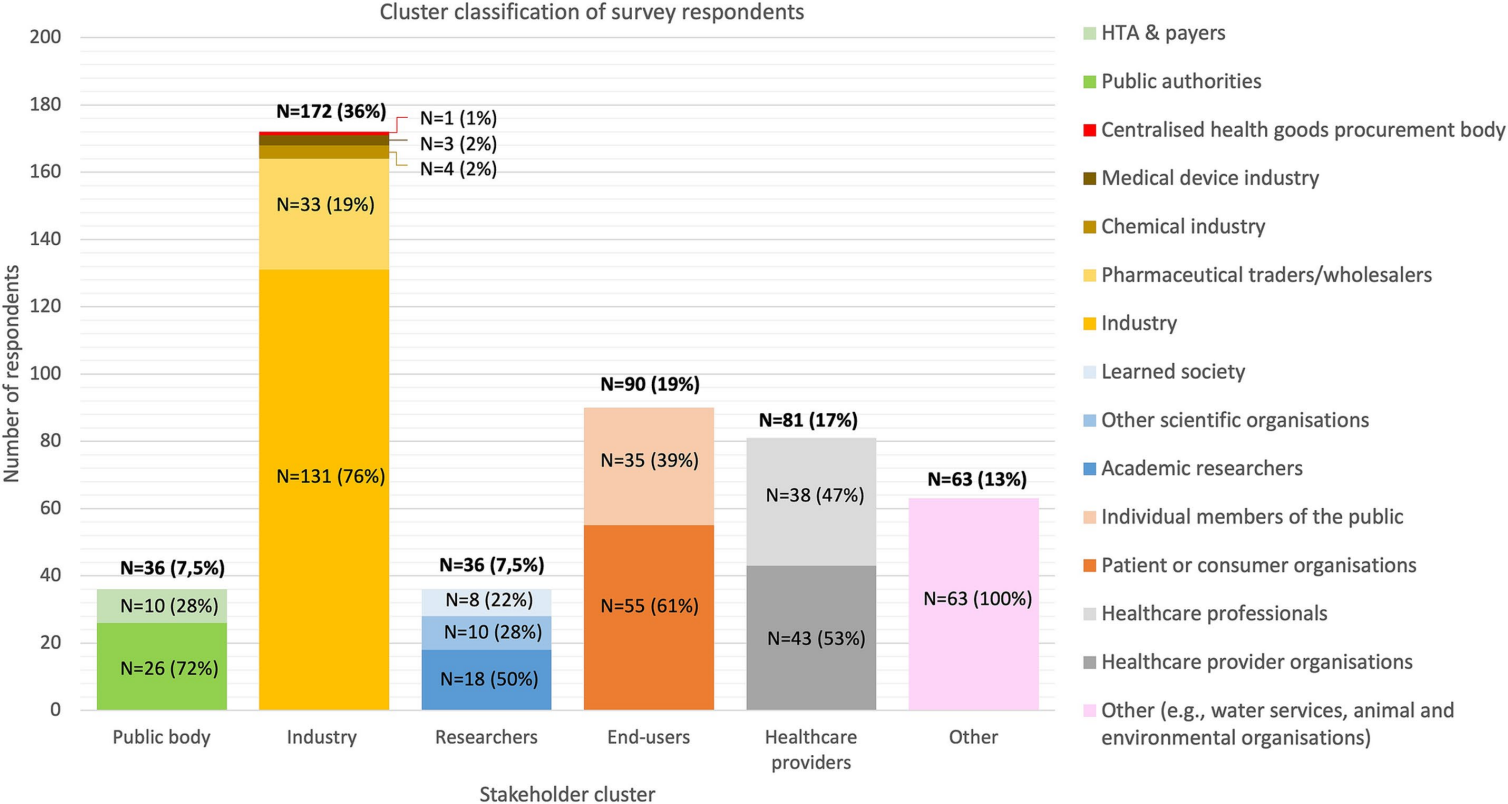
Distribution of the survey respondents by stakeholder group and subgroup classification. Stakeholders that did not identify with one of the above-mentioned clusters were assigned to the “other” cluster. HTA, Health Technology Assessment body.

A total of 478 responses on the public consultation survey were received. The industry group was the largest representing up to 36% of the total number of respondents, followed by end-users (19%), healthcare providers (17%), researchers (7.5%), and public bodies (7.5%) (Figure 2).

### 3.1 Conflicting suggestions on general stakeholder perspectives related to the UMN concept: qualitative results

Via open answer boxes and open-ended questions, stakeholders highlighted that for many disease areas patients still face (high) UMN and noted that the current UMN definition and regulatory framework lack clarity and comprehensiveness. With respect to the UMN concept and its definition, stakeholders reported on three main aspects (i) scope, (ii) flexibility, and (iii) binary nature of the UMN concept. However, perspectives highly differed between stakeholder groups:

*(i) Scope of the UMN concept*: HCPs expressed concerns regarding the restrictive nature of the current UMN definition, emphasizing the need for a broader scope that considers factors beyond the availability of alternative treatments. Additionally, both researchers and HCPs highlighted the importance of expanding the definition to include diagnostics and HCPs also suggested including supply problems. Rapid and accurate diagnostics were deemed essential for effective healthcare delivery and challenges related to medication supply disruptions were recognized by HCPs as significant contributors to UMN.

*(ii) Clarity of the UMN concept*: HCPs and researchers cautioned against a rigid UMN definition and one-size-fits all approaches, with strict pre-defined eligibility criteria, suggesting a flexible, multi-stakeholder-endorsed approach to better address healthcare complexities. Conversely, others (i.e., public bodies, industry) emphasized the necessity of clear, quantifiable criteria in a structural framework to guide innovation and address evidence gaps, advocating for an adaptable definition that evolves over time. Proposals and reflections on these UMN criteria are discussed under 3.2.

*(iii) Binary approach to the UMN concept*: Acknowledging the nuanced nature of UMN, public bodies and end-users emphasized the need for a non-binary approach that quantifies different levels of need. Suggestions included grading UMN based on severity and prioritizing incentives, accordingly, thereby accommodating the diverse healthcare landscape and varying degrees of need across different disease areas. Stakeholders underscored the dynamic nature of UMN, advocating for an adaptable definition that evolves over time to reflect changing healthcare priorities and emerging needs. This approach emphasizes flexibility and responsiveness.

### 3.2 Stakeholder perspectives on the proposed UMN definition, its criteria and respective implementation in the EU legislative proposal

#### 3.2.1 Quantitative results and the respective implementation in EU legislative proposals

**Figure 3:**
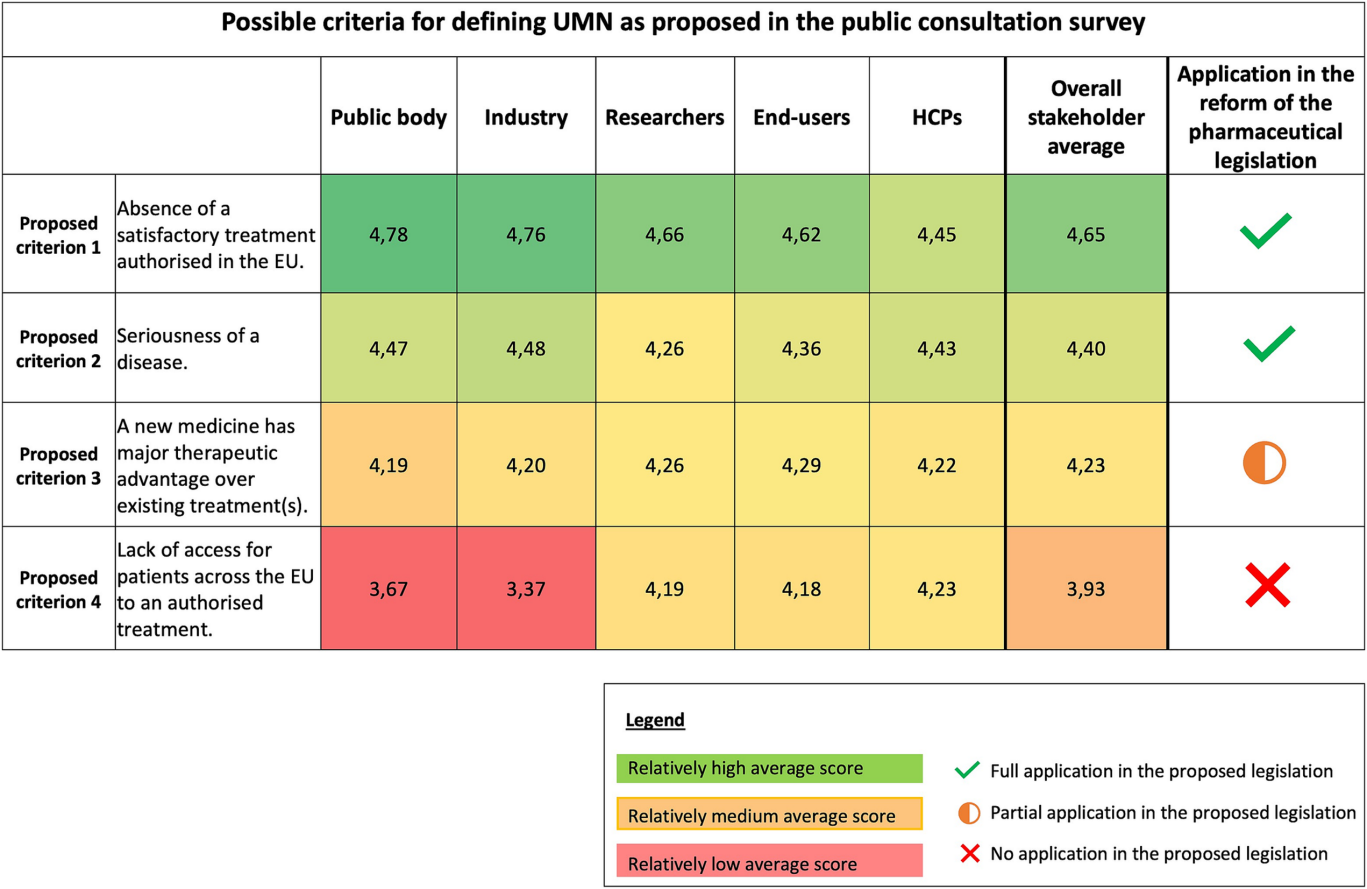
Heatmap of quantitative stakeholders’ ratings of proposed unmet medical need criteria. Average scores range from 1 to 5 and were colored relatively to another using the conditional formatting tool in Excel. The highest average scores are indicated in green; the lowest average values are indicated in red, and the values in between are colored in orange/yellow. EU, European Union; HCPs, Healthcare providers; UMN, Unmet medical need; QoL, Quality of life.

In the public consultation survey, the EC proposed four criteria to be potentially included in the UMN definition: (i) absence of a satisfactory treatment authorized in the EU, (ii) seriousness of a disease, (iii) major therapeutic advantage over existing treatment(s), and (iv) lack of access for patients across the EU to an authorized treatment. Quantitative survey question analysis (Figure 3) showed that stakeholders considered the following criteria as the most important criteria to define UMN: (i) the absence of satisfactory treatment authorized in the EU and (ii) the seriousness of the disease. For the other two proposed criteria the opinions are relatively less favorable; the public bodies and industry stakeholder group indicating on average relatively lower importance for the criterion on lack of access for patients across the EU to an authorized treatment.

**Figure 4:**
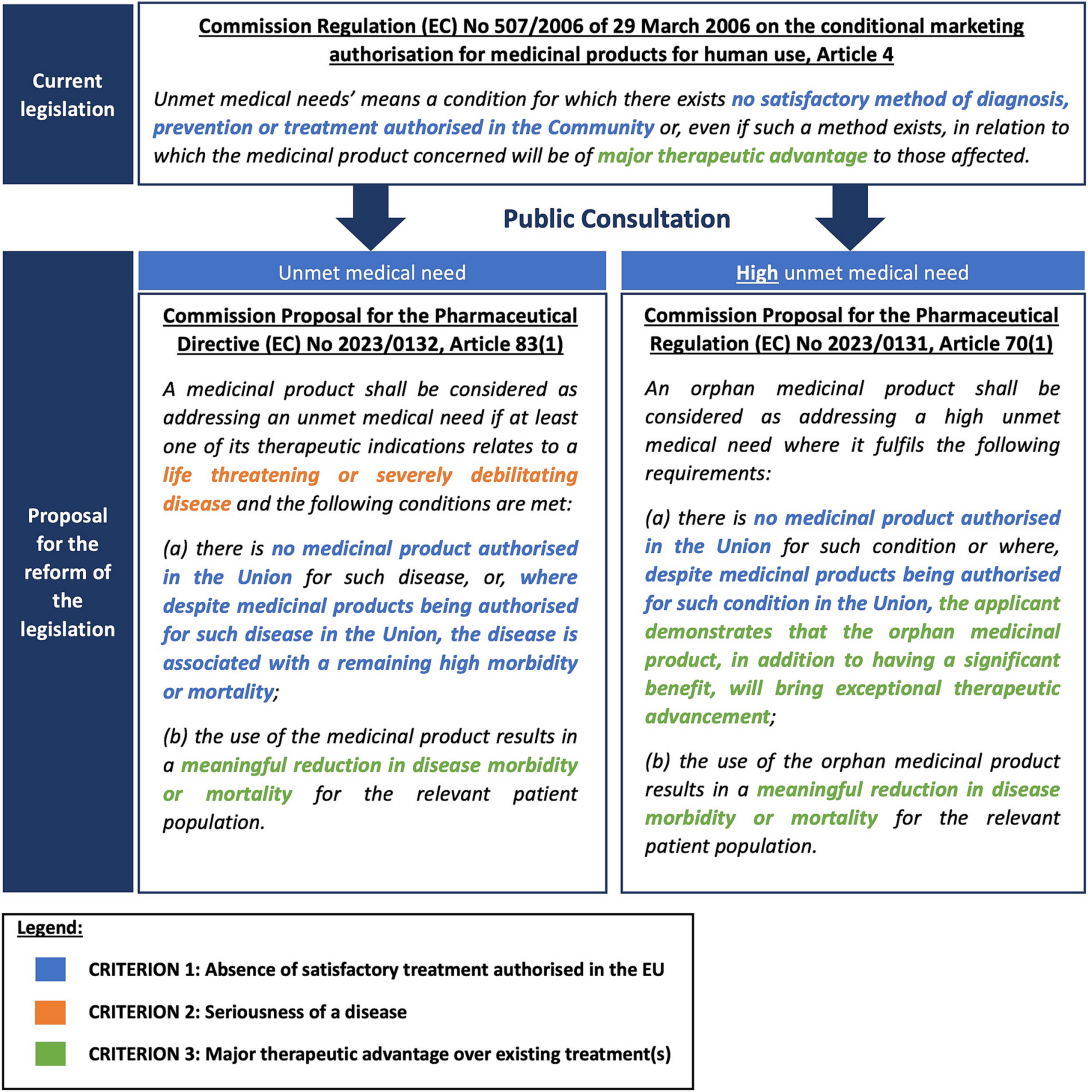
The definition of (high) unmet medical need in the existing and proposed pharmaceutical legislation.

Figure 4 provides an overview of the definition of UMN included in the existing pharmaceutical Regulation [(EC) Regulation No. 507/2006], concerning conditional marketing authorization, and the proposed definition included in the proposal for the reform of the pharmaceutical legislation published in April 2023 (Figure 4). The revised legislative package introduces two definitions: one for regular UMN (Proposal for a Directive 2023/0132) and another for orphan medicinal products (Proposal for a Regulation 2023/0131), distinguishing between UMN and high UMN, respectively.

As indicated in Figure 4, both the criterion on the absence of a satisfactory treatment authorized in the EU (Proposed criterion 1) and the major therapeutic advantage over existing treatments (Proposed criterion 3) were retained in the new legislative proposal. The wording for the proposed criterion 3 was updated from “be of major therapeutic advantage to those affected” to “results in a meaningful reduction in disease morbidity or mortality for the relevant patient population.” In addition to these two criteria, the seriousness of the disease (Proposed criterion 2) was included in the new legislative proposal. This criterion only applies to the regular UMN definition [Proposal for a Directive 2023/0132] and is not included in the orphan definition of high UMN [Proposal for a Regulation 2023/0131]. One of the proposed criteria, the lack of access (Proposed criterion 4), was excluded from the proposed definition.

#### 3.2.2 Qualitative results and the respective implementation in EU legislative proposals

**Figure 5:**
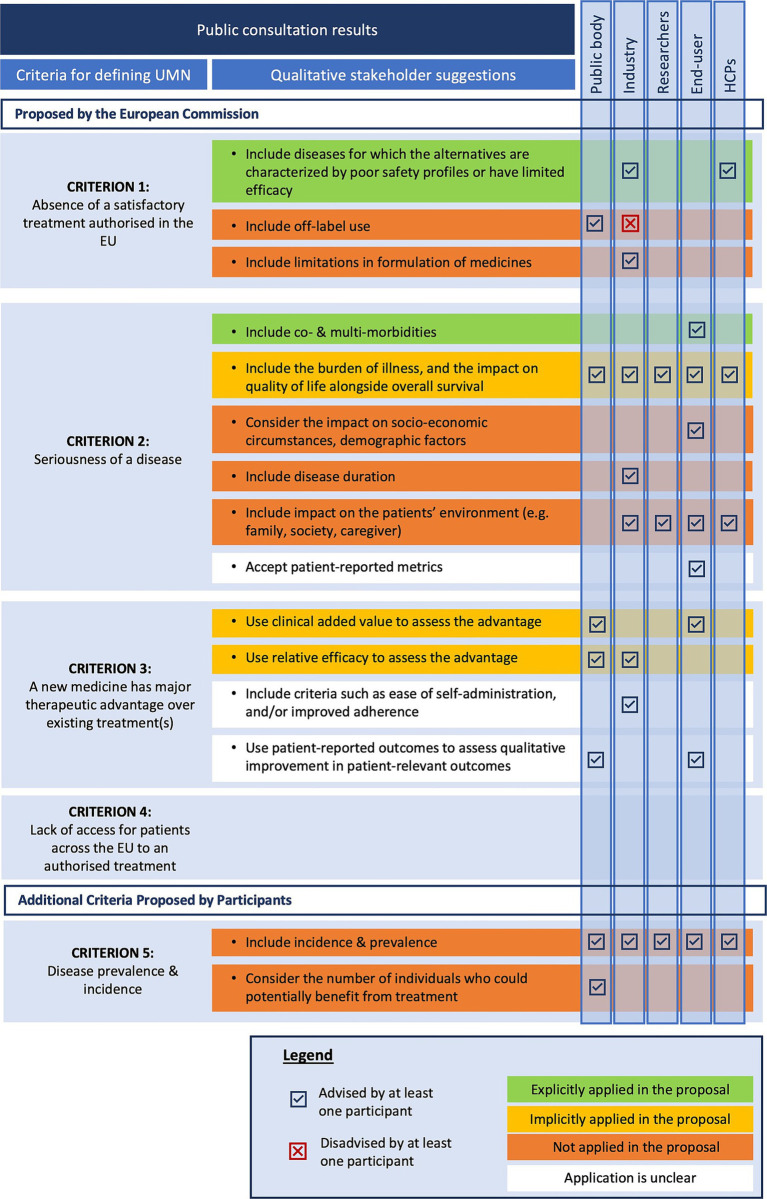
Stakeholder suggestions on unmet medical need criteria versus the legislative changes in the reform proposal. EU, European Union; HCPs, Healthcare providers; UMN, Unmet Medical Need.

In addition to the closed multiple-choice questions, participants were given the opportunity to provide complementary input regarding the proposed criteria. Figure 5 presents an overview of the additional qualitative suggestions offered by participants in the public consultation survey related to the proposed UMN criteria.

##### 3.2.2.1 Proposed criterion 1: absence of a satisfactory treatment authorized in the EU

Both industry representatives and HCPs stressed the importance of addressing diseases where existing medication has poor safety profiles or limited efficacy in some subpopulations. This suggestion is reflected in the new proposed UMN definition since medicines addressing diseases with a remaining high morbidity or mortality are still considered targeting an UMN despite the presence of alternative medicines. Public bodies advocated considering off-label use as part of alternative treatments, while industry respondents argued against including off-label treatments since they believe these treatments lack established safety and efficacy. The proposed UMN definition excludes off-label use, focusing solely on medicines authorized in the European Union. Some industry respondents suggested broadening the definition to include formulations with poor pharmacokinetic profiles, but this suggestion has not been explicitly adopted. It is unclear whether pharmacokinetic improvement would be considered as an exceptional therapeutic advancement (a criterium in the high UMN definition of the Regulation, see Figure 4).

##### 3.2.2.2 Proposed criterion 2: seriousness of the disease

Six suggestions could be identified based on the stakeholder responses to the public consultations. First, end-users proposed to include co- and multi-morbidities in the consideration of UMN. Second, most respondents suggested incorporating the burden of illness and its impact on patients’ quality of life (QoL) alongside overall survival when assessing disease seriousness within specific patient populations. However, these considerations are not explicitly included in the proposed definition. Nevertheless, there is implicit recognition in the legislative proposal that diseases severely impacting QoL and burden of illness could be categorized as severely debilitating. The phrase “remaining high morbidity” may also relate to QoL. Beyond the direct health impacts, several other factors affecting patient QoL were noted by stakeholders. Third, end-users highlighted socioeconomic circumstances, and demographics as important QoL indicators. The integration of these aspects into the new UMN definition remains unclear.

Fourth, industry representatives advocated for including disease duration as a criterion for assessing disease seriousness, which is currently absent from the proposed UMN definition. In addition to patient QoL, industry, end-user, researcher, and HCP participants stressed that seriousness should encompass not only the impact on the patient’s life but also on their broader environment (e.g., family, society, caregivers). Moreover, industry participants highlighted the need to consider financial impacts on families and caregivers, including indirect costs such as caregiving services and lost income. Sixth, end-users emphasized the importance of using patient-reported metrics to evaluate disease seriousness, though this is not explicitly mentioned and may have been included under the legislation without specific reference.

##### 3.2.2.3 Proposed criterion 3: a new medicine has major therapeutic advantages over existing treatments

Respondents emphasized the necessity for a clear understanding of the terminology “major therapeutic advantage.” Although this term is not literally used in the proposed UMN definition, it may be implicitly covered via the terminology “meaningful reduction in disease morbidity or mortality” in part (b) of the definition, which is slightly more concrete and hence partly addresses this reported concern. This terminology, and more specifically the word “meaningful,” could potentially point at the perception and experience of patients, which could potentially address the suggestions from public bodies and end-users to incorporate improvements in patient-relevant outcomes. In this regard, end-users underlined the importance of patient involvement and using patient-experience data to gain insight into the “true” benefit that a particular medicine might bring to patients. However, the proposed legislation does not explicitly state whether patient-reported outcomes will be utilized for this assessment. Furthermore, the inclusion of criteria such as ease of self-administration, and improved adherence to assess therapeutic advantage remains unclear in the proposed UMN definition.

##### 3.2.2.4 Proposed criterion 4: lack of access for patients across the EU to an authorized treatment

For “lack of access for patients across the EU to an authorized treatment” no specific additional qualitative suggestions were formulated apart from reflections on the relevance of this criterion. For instance, HCPs and industry respondents expressed concerns about including this criterion in the UMN definition, arguing that access issues are primarily due to economic decisions by Member States or pharmaceutical companies. They warned against attributing lack of access to patients as a criterium, as it is often influenced by national responsibilities and payment systems.

##### 3.2.2.5 Additional proposed criteria: disease prevalence and incidence

For every stakeholder group at least one respondent emphasized the significance of incorporating disease prevalence and incidence rates into the definition of UMN. More specifically, public bodies suggested considering the number of individuals who could potentially benefit from treatment, highlighting the importance of understanding the epidemiological landscape of the disease. Despite these suggestions, disease prevalence and incidence are not included in the proposed UMN definition in the legislation.

### 3.3 Stakeholder perspectives on the proposed incentive measures to drive R&D in UMN-areas and its respective implementation in the EU legislative proposal

#### 3.3.1 Quantitative results and the respective implementation in EU legislative proposals

The EC proposed in the public consultation survey seven incentive measures to foster innovation and potentially encourage companies to focus R&D in disease areas with (high) UMN. These proposals included (1) public listing of priority therapeutic areas, (2) early scientific support and expediting measures for review/authorization, (3) maintaining current market and data protection periods, (4) introducing new incentives on top of the current regulatory protection periods, (5) providing different regulatory protection periods depending on the medicines’ purpose, (6) reducing the current regulatory protection periods and (7) requiring transparent reporting from companies on R&D costs and received public funding.

**Figure 6:**
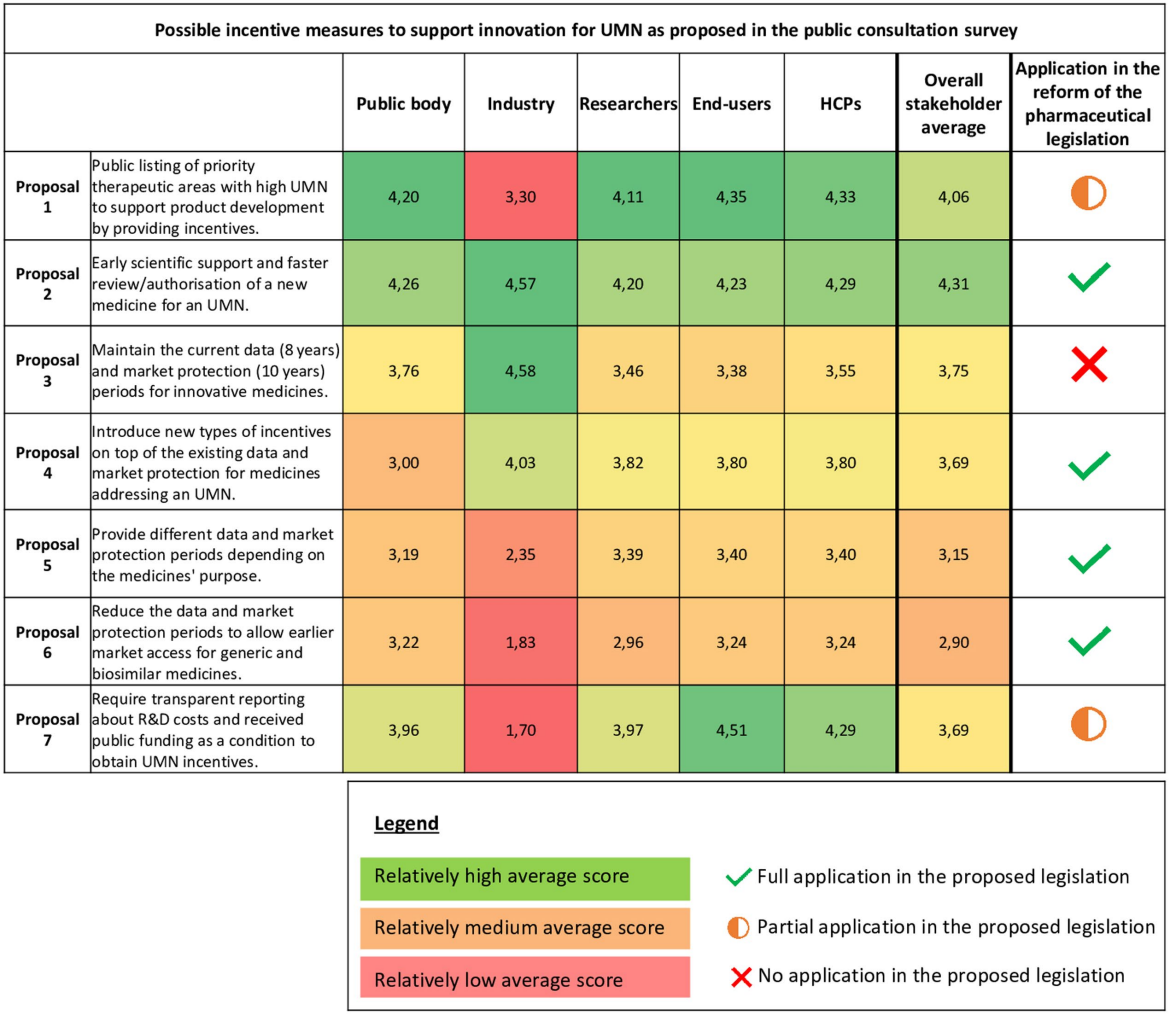
Heatmap describing stakeholders’ responses on the proposed incentive measures for unmet medical need. Average scores range from 1 to 5 and were colored relatively to another using the conditional formatting tool in Excel. The highest average values are indicated in green; the lowest average values are indicated in red, and the values in between are colored in orange/yellow. UMN, unmet medical need; HCPs, Healthcare providers; QoL, Quality of life.

The quantitative survey analysis (Figure 6) showed that on average, proposal 1 and 2 were relatively most welcomed by stakeholders. While the proposed incentive measures regarding regulatory protection periods (proposal 3, 4, 5, 6) were considered relatively less favorable by most stakeholders, industry respondents in particular indicated to be strongly in favor of maintaining or receiving additional regulatory protection periods for medicines targeting an UMN (proposal 3, 4). Moreover, industry was the only stakeholder group that responded negative to the proposal for enhancing transparency on R&D costs and received public funding for developing novel medicines addressing UMN (proposal 7).

#### 3.3.2 Qualitative results and the respective implementation in EU legislative proposals

**Figure 7:**
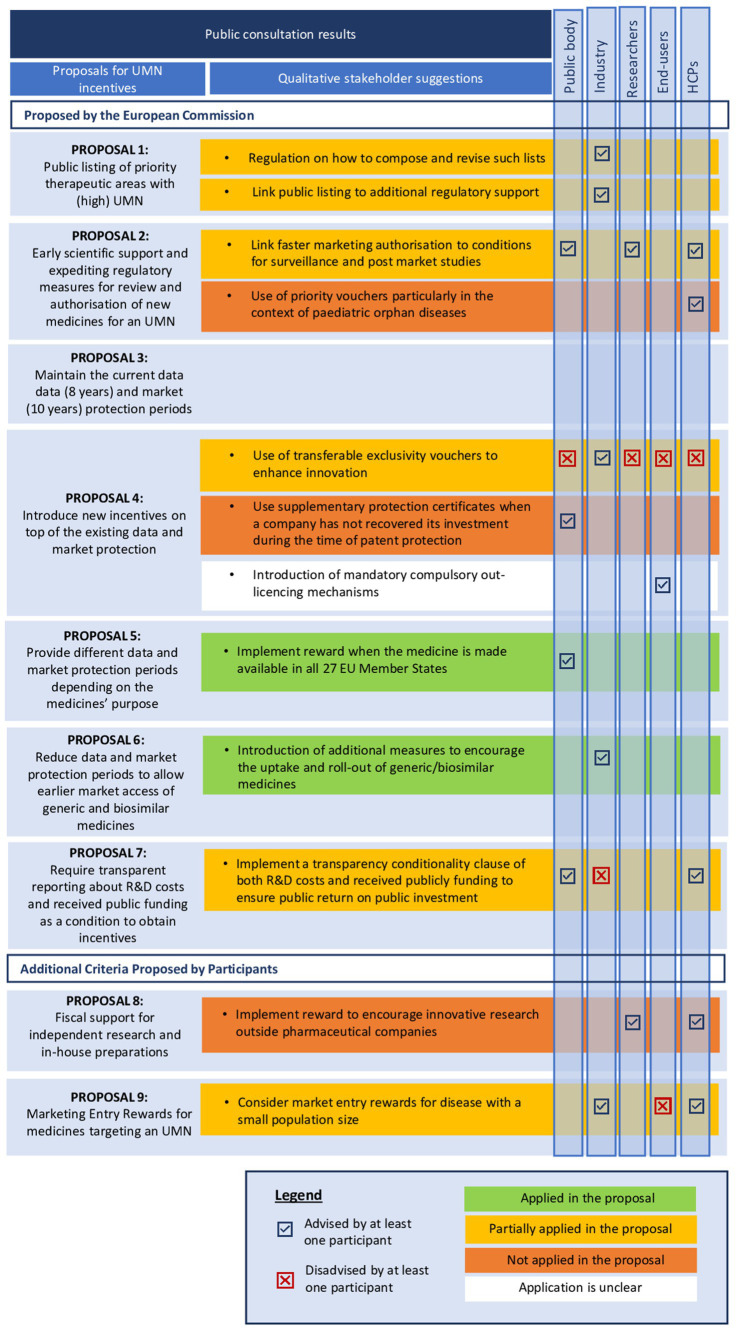
Qualitative insights from stakeholders on the UMN-related incentive measures. UMN, unmet medical needs; EU, European Union; IPR, intellectual property rights; RDP, regulatory data protection; R&D, research and development.

In addition to the closed multiple-choice questions, participants had the possibility to provide complementary input regarding the proposed incentive measures outlined in the public consultation survey. Figure 7 presents an overview of the additional qualitative suggestions offered by participants related to the UMN incentives.

##### 3.3.2.1 Proposal 1: public listing of priority therapeutic areas

While most stakeholders were strongly in favor of developing public listings of priority therapeutic areas, industry respondents in particular stressed that more regulation is needed on how such priority lists are being composed and revised. This proposed incentive measure was partially implemented in the proposal for the revised EU pharmaceutical legislation, limiting its scope to antimicrobials [Proposal for a Regulation (EC) No 2023/0131, Art. 40(3,4)]. More specifically, the legislative proposal refers to the WHO’s priority pathogen list and summary report describing the most pressing antibiotic-resistant pathogens as well as the methodological approach for developing the priority list. Industry respondents also emphasized that, to ensure a successful application, public listings for priority therapeutic areas should be combined with additional regulatory support for drug developers. This suggestion was partially implemented in the revised EU legislation [Proposal for a Regulation (EC) No 2023/0131, Art.60 (1), Art.89], given that antimicrobials are considered an area of UMN and thus, companies and not-for-profit organizations conducting R&D for priority pathogens are entitled to receiving (i) enhanced scientific and regulatory support and (ii) accelerated regulatory assessments, as discussed in proposal 2.

##### 3.3.2.2 Proposal 2: scientific advice and expediting regulatory measures for review and authorization

In the existing EU legislation, the UMN definition is officially used as an eligibility criterion in the context of conditional marketing authorization [Regulation (EC) No 507/2006, Art. 4] and implicitly in the context of the orphan designation [Regulation (EC) No 141/2000, Art. 3]. In the proposed reform, UMN as an eligibility criterion is explicitly extended to applications in the PRIME scheme and accelerated assessment [Proposal for a Regulation (EC) No 2023/0301, Art. 60]. This extended application of the UMN concept in these particular regulatory mechanisms corresponds with stakeholders’ perspectives, as they were generally in favor of the measure to enhance scientific and regulatory support. However, both public bodies and HCPs emphasized that such incentives must still be applied with vigilance in practice. For example, they warned that these measures may primarily increase the risk of allowing products of uncertain value to the market. Furthermore, they stressed that the assessment of medicines’ safety, quality and efficacy should not be shifted from pre- to post MA to the detriment of patients, therefore suggesting linking the implementation of such measures to conditions for surveillance and post-market studies. These suggestions were partially included in the proposal for a regulation (EC) No 2023/0131 by allowing EMA to impose additional post-marketing studies on companies, if necessary, to evaluate medicines’ safety and efficacy. The suggestion of end-users to use priority vouchers for rare pediatric diseases was not applied in the revised pharmaceutical legislation. Although innovative medicines with orphan designation are considered to address an UMN and are eligible for (i) enhanced scientific and regulatory support and (ii) accelerated regulatory assessments, these incentives are not equal to the concept of priority vouchers.

##### 3.3.2.3 Proposal 3–6: incentive measures related to IPR and RDP for medicines targeting UMNs

Industry respondents emphasized the need for maintaining the current regulatory and data protection periods for innovative medicines or providing additional incentives on top of the current regulatory and data protection periods to foster innovation. In this regard, the EC proposed to introduce a transferable exclusivity voucher (TEV) as an additional incentive, which means either granting manufacturers an extra year of data exclusivity on any one of the medicines in their portfolio or allowing them to sell the voucher to other developers. Stakeholders had rather conflicting opinions on this proposal. Whilst industry respondents were strongly in favor of this measure, public bodies, end-users, and HCPs believed this measure would cause overcompensation for pharmaceutical developers. The use of TEVs was partially included in the revised legislation, yet its applicability is restricted to the field of antimicrobials, encouraging the development of novel antibiotics to address the issues of AMR [Proposal for a Regulation (EC) No 2023/0301, Art. 40]. The suggestion of public bodies to introduce supplementary protection certificates as additional incentive for companies to ensure return of investment was not included in the proposal of the reform of the pharmaceutical legislation. Lastly, it remains unclear whether and how the suggestion of end-users to introduce mandatory compulsory out-licensing mechanisms was integrated in the revised legislation.

The reform of the EU pharmaceutical legislation includes a gradual incentive structure allowing pharmaceutical developers to receive additional regulatory protection for medicines targeting an UMN. For example, the proposal for a Directive (EC) No 2023/0302. Art. 80(2)—Art. 81(1,2) includes a reduced standard data protection period for medicines from 8 to 6 years. However, data protection periods may be prolonged with (i) +24 months when medicinal products are released in all 27 EU Member States and continuously supplied or (ii) +6 months when the medicinal product addresses an UMN. Moreover, whilst the regular period for marketing exclusivity is 9 years for orphan medicines, pharmaceutical developers can receive an extra year of marketing exclusivity (10 years) when an orphan medicine addresses a high UMN [Proposal for a Regulation (EC) No 2023/0301, Art. 71(1); Art 72(1,2)]. This gradual incentive structure for regulatory protection periods corresponds with public bodies suggestions to maintain a binary approach for UMN incentives and determine regulatory protection periods based on (i) the degree of UMN (i.e., high or not) and (ii) the extent to which a treatment meets patients UMN, referring to the medicines’ scientific efficacy as well as its availability across the Union.

##### 3.3.2.4 Proposal 7: transparency on R&D costs and received public funding as a conditionality clause

The proposal for making UMN-related incentives contingent on greater R&D transparency for drug developers was welcomed by end-users, HCPs and public bodies, as they suggested introducing a conditionality clause on transparency of both R&D costs and received public funding to ensure public return on public investment. In contrast, industry representatives stressed that obligating transparency on R&D costs and public funding as a condition to obtain incentives would increase the burden on companies and would set barriers to innovation. As a result, the EC partially applied this suggestion in the reform of the EU pharmaceutical legislation yet limiting its scope to solely requiring pharmaceutical companies to report any directly received public funding [Proposal for a Directive (EC) No 2023/0302, Art. 57(1)]. Furthermore, the EC did not make this transparency clause a condition for eligibility for UMN incentives.

##### 3.3.2.5 Additional proposed incentive measure by stakeholders: financial support

Both HCPs and researchers pointed out that the focus should not be primarily on introducing additional incentives for industry but rather on funding (i) independent academic R&D and (ii) in-house hospital preparations for particular treatments. End-users agreed with these suggestions, underlining the importance of allocating more public funding to hospitals and academic research to tackle the issues of academic knowledge commercialization. However, these suggestions were not mentioned in the proposal for the reform of the EU pharmaceutical legislation.

##### 3.3.2.6 Additional proposed incentive measure by stakeholders: marketing entry rewards

Industry representatives highlighted that the lack of reimbursement for many innovative drugs makes it difficult to meet patients’ needs in practice and suggested that, in those disease areas for which the target population is rather small, new pricing mechanisms (e.g., de-linkage payment models) and additional market uptake or entry rewards such as lump sums could further support innovation. HCPs agreed on this, adding that HTA bodies should give preference to therapies targeting an UMN. However, the proposal for the reform of the EU pharmaceutical legislation did not include any specifications on the introduction of marketing entry rewards (MER) as an incentive to stimulate innovation, which is consistent with the views of end-users who stated that HTA bodies should maintain stringent standards for newly authorized therapies, especially when the added therapeutic value seems to be marginal or negligible.

## 4 Discussion

Upon the publication of the proposed reform of the EU pharmaceutical legislation, stakeholders across the drug development landscape have argued against the proposed criteria related to the UMN definition and the associated incentives (12, 13). To better understand how the public consultation has informed the current legislative proposal, this study provides an in-depth analysis of stakeholder responses to the public consultation of the proposal for the reform of the EU pharmaceutical legislation, that covered proposed criteria and incentives for UMN to inform the general EU pharmaceutical legislation, and assessed how stakeholders’ perspectives and recommendations were implemented in the current proposal for the reform of the EU pharmaceutical legislation. This analysis focuses specifically on the definition of UMN and incentive measures to stimulate innovation and development in disease areas with (high) UMN and offers an academic perspective, employing rigorous methodological analysis.

Regarding the UMN definition, this comprehensive analysis of stakeholder responses highlighted three key recommendations: (i) extending the scope of the definition beyond pharmaceutical developments, (ii) ensuring sufficient flexibility, and (iii) approaching UMNs in a non-binary way. All respondents agreed that the absence of a satisfactory treatment authorized in the EU and the seriousness of a disease are the most important criteria, which were subsequently included in the proposed legal definition of UMN. Additionally, the criterion of a major therapeutic advantage over existing treatments is a criterion in the existing UMN definition [Regulation (EC) No 507/2006, Art. 4], but it was not as such included in the new legislative proposal. However, this criterion is rather implicitly included in the reform of the EU pharmaceutical legislation and rephrased as (i) “the medicinal product results in a meaningful reduction in disease mortality or morbidity” in case of the UMN definition [Directive (EC) No 2023/0132, Art. 83(1)] or (ii) “the applicant demonstrates that the orphan medicinal product, in addition to having a significant benefit, will bring exceptional therapeutic advancement” in the definition for high UMN [Regulation (EC) No 2023/0131, Art. 70(1)]. While disease incidence and prevalence were also qualitatively suggested by some stakeholders, these criteria were not included in the reform of the EU pharmaceutical legislation.

Regarding UMN-related incentives, this comprehensive analysis of stakeholder’s responses highlighted two major legislative changes in the proposal for the reform of the EU pharmaceutical legislation: (i) a reduction in regulatory data protection from 8 to 6 years [proposal for a Directive (EC) No 2023/0302. Art. 80(2)—Art. 81(1,2)], and (ii) the introduction of TEVs for antimicrobials [Proposal for a Regulation (EC) No 2023/0301, Art. 40]. While most industry participants opposed changes to the regulatory data protection periods, they supported the introduction of TEVs. In contrast, other stakeholder groups were hesitant to provide any additional incentives, including RDP and transferable exclusivity vouchers, and instead recommended focusing on scientific support and expedited regulatory measures. Subsequently, the revised legislative package extended the application of the UMN concept to the PRIME scheme and accelerated assessment [Proposal for a Regulation (EC) No 2023/0301, Art. 60].

### 4.1 Enhanced clarity on UMN criteria

While this study highlighted the need for clarity from both industry and public bodies regarding the eligibility criteria for UMN, many of these criteria in the current legislative proposal remain open to interpretation. This aligns with suggestions from HCPs and researchers to maintain a flexible and dynamic approach to the UMN concept. However, the European Federation of Pharmaceutical Industries and Associations (EFPIA) warns that less ambiguous criteria could lead to uncertainty for medicine developers, especially in areas reliant on incremental innovation (12). For instance, the proposed criterion of “severely debilitating” raises questions about measurement and cut-offs (12, 14). EFPIA’s assessment indicates that most medical products could be considered life-threatening or seriously debilitating, necessitating clearer criteria to enhance predictability (12).

Another ambiguous criterion is “remaining high morbidity or mortality” (12). Similarly, the criterion of “meaningful reduction in disease morbidity and mortality” is seen by EFPIA as challenging and unpredictable due to the underlying value judgment and the implied need for comparative clinical data (12). In the context of orphan medicines, the additional criterion of “exceptional therapeutic advancement” creates uncertainty about its definition, potentially hampering innovation in rare diseases, where only 6% of known rare diseases have an approved treatment (12, 14). Because of this, The European Patient Forum calls for a universally accepted definition of “added therapeutic value,” stressing that systematic patient involvement is key to obtain a comprehensive understanding on a medicines’ true benefit–risk balance (10). The authors of this study, also question whether there is a meaningful difference in the high UMN definition between “exceptional advancement” and “meaningful reduction,” [Regulation (EC) No 2023/0131, Art. 70(1)] and whether the latter is necessary since it is already part of the UMN definition, which is required for high UMN eligibility.

EFPIA argues that this unpredictability could hamper investments and raises concerns about scenarios where high uncertainty persists at the time of approval, a common issue for orphan medicinal products (12). The authors of this study stress that the primary goal of the UMN definition is to stimulate research and development in areas where investments or therapeutic advancements are currently lacking or limited by linking it to incentives, such as extended RDP. However, predictability is crucial for the pharmaceutical industry; without it, any incentives linked to the UMN definition will be insufficient to make a significant impact and support research in these areas. The authors also believe that UMN will evolve over time, and since pharmaceutical research and development is a lengthy process, the concept must be flexible to accommodate changes over time. Too restrictive criteria can therefore hamper innovation, which is highly unfavorable. A potential solution is to develop frameworks for the identification of needs, such as the one proposed by the Belgian knowledge center (KCE). These results can further support decision-makers in allocating incentives to the appropriate products.

### 4.2 Increased focus on quality of life and patient involvement

There has been growing attention to the impact of QoL as an outcome measure to evaluate the value of medicinal products, rather than just traditional clinical outcomes such as overall survival or mortality (15–17). Moreover, patient involvement in clinical research has gained importance to ensure that what truly matters to patients is measured through patient-relevant outcome measures (18). This patient-centered focus was reflected in the qualitative stakeholder responses, where multiple participants underscored the importance of patient involvement and QoL in assessing the satisfaction with existing alternatives and the seriousness of the disease. Patient experiences are becoming increasingly important, as confirmed by the EMA through the qualification of a framework for patient preference studies (19). This evolution is reflected in the new proposed legislative definition for UMN by integrating the criterium morbidity and including the term “meaningful,” which implies a value judgment and the possibility to include patient perceptions. However, the European Patients Forum (EPF) argues that considering only “mortality and morbidity” is too restrictive, as it ignores other important life-changing indicators. They propose including the impact on QoL more explicitly and involving patients in the definition’s development (13).

### 4.3 Modulation of UMN

The suggestion to move away from a binary approach for the UMN definition has been partially addressed. Some gradation is possible in the context of orphan medicinal products, with a distinction made between UMN and high UMN based on whether there is proof of “exceptional therapeutic advancement.” However, questions remain about how this will be demonstrated, and which methods are to be used. EURORDIS, the umbrella patient organization for rare diseases in Europe, requests more clarity on how patient representatives will be involved in regulatory practices (14), a point also noted by EPF (13). This two-level approach partially meets the proposal for a modular system and is welcomed by EURORDIS (14), but it could be extended to a three-level scale: high, medium, and large UMN, as proposed by Horgan et al. (16).

### 4.4 Incentives to drive R&D in UMN-areas

On the one hand, UMN is in some studies found to be one of the most influential drivers in pharmaceutical sciences (20, 21). On the other hand, factors like market size, scientific grounds, expected return on investment, and historical funding often outweigh the remaining burden of disease in funding decisions (22–25). As a result, the proposed reform of the EU pharmaceutical legislation aims to enhance innovation in areas of UMN (2).

Nevertheless, the European industry organization, EFPIA, warns that the proposed incentive framework will not suffice to create this shift (26). In order to generate real advances in UMN areas, EFPIA suggests additional legislative adjustments, such as the implementation of transferable exclusivity extensions and a predictable RDP system since they believe variable RDP periods based on the “purpose of the medicine” could undermine innovation in Europe (26). Nevertheless, this analysis shows that many other stakeholders are not in favor of extended RDP periods or IPR for companies. Furthermore, stronger pharmaceutical monopolies can increase drug prices and delay availability (27, 28). Therefore, balancing the stimulation of R&D with avoiding monopolies that disrupt the R&D system is crucial.

Besides adjustments in RDP, EFPIA also proposes extending the eligibility scope for the PRIME scheme and allowing earlier PRIME access (26). In its reaction on the proposal, EFPIA emphasizes the need for consistent and predictable application of the PRIME scheme (26). Besides regulatory pathways, adjustments to the orphan drug regulation are believed necessary to further enhance innovation (27, 28).

Lastly, it must be emphasized that basic research is vital for pharmaceutical development, often starting in early research settings (20). Moreover, research shows that developing treatments in non-profit or academic settings could be a viable alternative when EFPIA companies face insufficient incentives to address UMNs (29, 30). Therefore, most stakeholders favor regulatory flexibility such as early scientific advice and faster reviews. Whilst academic-based drug development is becoming increasingly important to address the most persistent UMN, a study by Kallio et al. pointed out the lack of knowledge and skills of academia within the regulatory environment (31). The lack of clear and transparent communication between stakeholders (i.e., academia and authorities) poses a significant barrier for supporting academic development, underlining the need to raise awareness of available regulatory support tools and training to foster academic drug development.

The European Cancer League underscored that enhanced regulatory support alone is not sufficient to foster academic-based drug development, emphasizing the need for (i) non-commercial registration trajectories for marketing authorizations and (ii) public funding for breakthrough innovative medicines developed by academia (32). The latter is considered key in ensuring the translation of academic discoveries into targeted therapies, requiring further efforts in setting up multi-stakeholder partnerships to adequately address the highest UMN (33). One option is public-private partnerships, where academia drives innovation and industry provides resources, which are critical for fostering innovation (29). Moreover, for diseases like Alzheimer’s, the federal government is the largest public funder of research, while the pharmaceutical industry focuses on late-stage drugs (34, 35). Additionally, diseases in high-income countries receive significantly more research attention compared to those in low-income countries, a disparity that regulatory incentives alone cannot address (23). Therefore, expanding support for these initiatives could complement industry efforts, ensuring UMN are addressed even when traditional market-driven incentives fall short.

### 4.5 Incentivizing equitable access for medicines targeting UMN

When a medicine is eligible for UMN incentives at the European level, it does not guarantee patient access across EU member states. In most member states, extensive pricing and regulatory procedures must be initiated following the submission by the marketing authorization holder (MAH). Currently, there is no obligation for companies to make the drug available in any country upon authorization, leading to reported inequalities in medicine availability across the EU (36–38), with later launches in member states with lower GDP (36).

A key challenge remains achieving alignment across organizations and member states. Currently, there is a lack of consensus between the EMA and national HTA bodies or payers on the UMN concept (5). Although the revised EU legislation aims to enhance and align this understanding among stakeholders, it does not provide concrete guidance on implementation. Therefore, it is still unclear how national HTA bodies and payers will handle medicines that the EMA perceives as targeting a UMN.

Although the proposed criterion on market access in European Member States was not explicitly included in the definition of UMN, it has been included as an eligibility criterion for add-on RDP period incentive. When medicinal products are made available in all 27 member states, the MAH can benefit a prolonged RDP period of 24 months. While this is a step toward achieving more equitable access to medicines in the EU, EURORDIS recommends developing a streamlined pathway that includes regulatory advice, marketing approval, and pricing and reimbursement activities at the EU level to allow early access to medicines for ultra-rare diseases (14). Similarly, in the context of the United Kingdom, the “Innovative Licensing and Access Pathway” (ILAP) was created to support and accelerate the development of medicines targeting UMNs and allows flexible support tools through the life cycle of medicines development using a multi-agency approach from regulators to HTA bodies (39). Alternative recommendations to stimulate earlier patient access include mandatory national pricing and reimbursement submission at the EU level, increased alignment on evidence requirements between the EMA and national payers, and enhanced and aligned national early access programs linked to European decisions (37, 40).

### 4.6 Strengths and limitations

One of the primary limitations of this study is the dynamic nature of the legislative landscape we are investigating. The laws and regulations under examination are currently in the revision process, and significant changes may occur before the final regulation and directive are adopted. This inherent uncertainty means that some findings and discussions presented in this study may become outdated or less relevant as the legislative process evolves. However, this evolving landscape also presents a unique strength. By analyzing the proposed revisions and stakeholder feedback during the public consultation, this research highlights critical topics and issues that are still under consideration. Our findings and discussions can influence ongoing debates and potentially shape the final content of the regulation and directive, providing valuable insights for policymakers, stakeholders, and researchers, and contributing to a more informed and nuanced legislative development process.

The conduct of both a quantitative and qualitative analysis of stakeholders’ responses on the proposed policy optimization measures as described in the public consultation on the Pharmaceutical Strategy to inform the reform of the pharmaceutical EU legislation, allows for an in-depth yet nuanced understanding of stakeholder’s perspectives. This approach ensures that agreements but most certainly discussion points among stakeholders on particular policy proposals are put into a broader context. It should be emphasized that stakeholders could voluntarily provide additional qualitative input to further clarify their answers in the public consultation survey. The voluntary nature of these qualitative data may result in a potential imbalance in perspectives among specific stakeholder groups, such as industry who provided substantial qualitative input compared to other stakeholder groups, making it at times difficult to draw general conclusions or find consensus across the diverse views represented. The cluster classification of stakeholder respondents provides the opportunity to make inter-group and between group comparisons of different stakeholder perspectives and allows for a more nuanced interpretation of group level viewpoints. However, it is important to note that the perspectives of stakeholders who self-identified as “other” (*n* = 63) were not included in this analysis. Although their insights could have been valuable to this study, given the heterogeneity of stakeholders in the “other” group, the authors anticipated that drawing generalizable conclusions from their responses would be challenging. The visualization of stakeholders’ views and their additional policy suggestions in heatmaps is an comprehensive approach to obtain a clear overview on general tendencies at stakeholder cluster level and allows to compare the different levels of satisfaction between stakeholders regarding the proposed policy measures. Moreover, the calculation of overall group averages indicates which measures received the highest score from all stakeholders and thus, were most widely supported.

An inductive coding approach was maintained for the qualitative analysis of stakeholder’s responses in the open answer text fields. As a consequence, there was a primary focus on topics/themes that were recurrently addressed by respondents in each stakeholder cluster. Therefore, the generalizability of the qualitative findings for all participants per stakeholder cluster should be carefully considered. Moreover, the classification of stakeholders into clusters was meticulously conducted in consultation with the entire research team, based on reported affiliations. Stakeholders who did not clearly align with any of the defined clusters were placed in the “other” category and subsequently excluded from the analysis. This approach results in a discrepancy with the stakeholder distribution used by the EC in their summary of results. However, this adjustment affects only 33 participants and is not expected to significantly influence the overall results. Finally, it should be noted that the quantitative analysis was conducted by one researcher, meaning that no cross-check of individual study results was performed. With respect to the qualitative analysis, inductive coding was performed by one researcher while analysis and synthesis were performed by two researchers.

## 5 Conclusion

This study provides a detailed analysis of stakeholder responses to the proposed EU pharmaceutical legislation revision, focusing on UMN definitions and incentives. Stakeholders proposed, in line with the proposed definition, to include disease seriousness and availability of alternatives in the UMN definition. Nevertheless, many stakeholders continue to highlight the ambiguity of the current definition and underscore a need for further discussion on the UMN definition. The distinction of UMN and high UMN within the legislative proposal was partially meeting the recommendation to apply a modular approach but could still be extended beyond orphan medicinal products. Industry participants opposed reducing RDP but supported transferable exclusivity vouchers as included in the legislative proposal, whereas other stakeholders preferred scientific and regulatory support over additional RDP incentives. The findings underscore the need for further discussion on UMN related incentives to stimulate innovation while ensuring patient-centric outcomes and equitable access to medicines across the EU.

## Data Availability

Publicly available datasets were analyzed in this study. This data can be found at: https://ec.europa.eu/info/law/better-regulation/have-your-say/initiatives/12963-Revision-of-the-EU-general-pharmaceuticals-legislation/public-consultation_en.

## Footnotes

^1^*Conditional marketing authorization* is a pragmatic tool for the fast-track approval of a medicine that addresses unmet medical needs of patients on the basis of less comprehensive data than normally required. The available data must indicate that the medicine’s benefits outweigh its risks, and the applicant should be in a position to provide the comprehensive clinical data in the future.
