# Supplementary material for: Unmet medical needs definition and incentives: stakeholders perspectives on the reform of the EU pharmaceutical legislation
Source: Front Med (Lausanne). 2025 Jan 7;11:1506243. doi: 10.3389/fmed.2024.1506243 (PMC11747691; doi:10.3389/fmed.2024.1506243)
Supplement: Supplementary file 1 [file Table_1.docx]

Supplementary Material

# Supplementary Tables

**Supplementary Table 1.** Public consultation survey of the European Commission containing multiple-choice and open answer questions with policy proposals related to the reform of the EU Pharmaceutical legislation.

| Open Public Consultation on the revision of the general pharmaceutical legislation |
| --- |
| **Introduction** |
| On 25 November 2020, the Commission published a Communication on a Pharmaceutical Strategy for Europe.  The Pharmaceutical Strategy identifies flagship initiatives and other actions to ensure the delivery of tangible results. As part of the implementation of the strategy, the Commission is evaluating the general pharmaceutical legislation^1^ and assessing the impacts of possible changes in the legislation as described in the relevant inception impact assessment.  This public consultation aims to collect views of stakeholders and the general public in order to support the evaluation of the existing general pharmaceutical legislation and the impact assessment of its revision. It builds further on the public consultation^2^ conducted for the preparation of the pharmaceutical strategy for Europe. The replies to that consultation will be taken into account for the revision of the general pharmaceutical legislation. The present questionnaire should be seen as a continuation of that process.  In parallel, the legislation for medicines for rare diseases and children is being revised as well. Separate consultation activities have been carried out for that revision.  This questionnaire is available in all EU languages and you can reply in any EU language. You can pause any time and continue later. You can download your contribution once you have submitted your answers.  A summary on the outcome of the public consultation will be published by the Commission services on the ‘ Have your say’ portal.  We thank you for your participation.  *[1]* ***Directive 2001/83/EC*** *of the European Parliament and of the Council of 6 November 2001 on the Community code relating to medicinal products for human use (OJ L 311, 28.11.2001, p. 67)* ***Regulation (EC) No 726/2004*** *of the European Parliament and of the Council of 31 March 2004 laying down Community procedures for the authorisation and supervision of medicinal products for human and veterinary use and establishing a European Medicines Agency (OJ L 136, 30.4.2004, p. 1)*  *[2] A report analysing the results of the pharmaceutical strategy consultation was published in November 2020.* |
| **About you** |
| 1. **Language of my contribution**    - Bulgarian    - Croatian    - Czech    - Danish    - Dutch    - English    - Estonian    - Finnish    - French    - German    - Greek    - Hungarian    - Irish    - Italian    - Latvian    - Lithuanian    - Maltese    - Polish    - Portuguese    - Romanian    - Slovak    - Slovenian    - Spanish    - Swedish |
| 1. **I am giving my contribution as**    - Academic/research institution    - Business association    - Company/Business organisation    - Consumer organisation    - EU Citizen    - Environmental organisation    - Non-EU citizen    - Non-governemental organisation (NGO)    - Public authority    - Trade Union    - Other |
| 1. **Which stakeholder group do you represent?**     - Individual member of the public    - Patient or consumer organisation    - Healthcare professional    - Healthcare provider organisation (incl. hospitals, pharmacies)    - Healthcare payer    - Centralised health goods procurement body    - Health technology assessment body    - Academic researcher    - Research funder    - Learned society    - European research infrastructure    - Other scientific organisation    - Environmental organisation    - Pharmaceutical industry    - Chemicals industry    - Pharmaceutical traders/wholesalers    - Medical device industry    - Public authority (e.g. national ministries of health, medicines agencies, pricing and reimbursement authorities)    - EU regulatory parner/EU institution    - Non-EU regulator/non-EU body    - Other (please specify) |
| 1. **First name:** [____________________] |
| 1. **Surname:** [____________________] |
| 1. **E-mail (this won’t be published):** [____________________] |
| 1. **Organisation name:** [____________________] |
| 1. **Organisation size**    - Micro (1 to 9 employees)    - Small (10 to 49 employees)    - Medium (50 to 249 employees)    - Large (250 or more) |
| 1. **Transparency register number:** [____________________] |
| 1. **Country of orgin:** [____________________] |
| The Commission will publish all contributions to this public consultation. You can choose whether you would prefer to have your details published or to remain anonymous when your contribution is published. For the purpose of transparency, the type of respondent (for example, ‘business association, ‘consumer association’, ‘EU citizen’) country of origin, organisation name and size, and its transparency register number, are always published. Your e-mail address will never be published. Opt in to select the privacy option that best suits you. Privacy options default based on the type of respondent selected. |
| 1. *** Contribution publication privacy settings**  - **Anonymous -** (Only organisation details are published: The type of respondent that you responded to this consultation as, the name of the organisation on whose behalf you reply as well as its transparency number, its size, its country of origin and your contribution will be published as received. Your name will not be published. Please do not include any personal data in the contribution itself if you want to remain anonymous). - **Public -** (Organisation details and respondent details are published: The type of respondent that you responded to this consultation as, the name of the organisation on whose behalf you reply as well as its transparency number, its size, its country of origin and your contribution will be published. Your name will also be published). - **I agree with the personal data protection provisions** |
| **Looking back** |
| As mentioned in the Inception Impact assessment, the revision aims to tackle the following problems:   - Unmet medical needs and market failures for medicines other than medicines for rare diseases and children; - Unequal access to available and affordable medicines for patients across the EU; - The current legislative framework may not be fully equipped to respond quickly to innovation; - Inefficiency and administrative burden of regulatory procedures; - Vulnerability of supply of medicines, shortages of medicines; - Environmental challenges and sustainability;   Any other issues, which might emerge from the evaluation. |
| **Q1: In your opinion, are there any other issues that should be addressed in this revision?**  [open answer field] |
| **Q2: How has the legislation performed in terms of the following elements?**   - Fulfilling its public health protection mission for patients and society.   - Very well   - Well   - Moderately   - Poorly   - Very poorly   - Don’t know - Promoting the development of new medicines, especially for unmet medical needs.   - Very well   - Well   - Moderately   - Poorly   - Very poorly   - Don’t know - Enabling timely development of medicines at all times, including during crises.   - Very well   - Well   - Moderately   - Poorly   - Very poorly   - Don’t know - Enabling timely authorisation, including scientific evaluation, of medicines in normal times.   - Very well   - Well   - Moderately   - Poorly   - Very poorly   - Don’t know - Enabling timely authorisation, including scientific evaluation during crises.   - Very well   - Well   - Moderately   - Poorly   - Very poorly   - Don’t know - Adapting efficiently and effectively to technological and scientific advancements and innovation.   - Very well   - Well   - Moderately   - Poorly   - Very poorly   - Don’t know - Ensuring medicines are of high quality, safe and effective.   - Very well   - Well   - Moderately   - Poorly   - Very poorly   - Don’t know - Addressing the competitive functioning of the market to support affordability.   - Very well   - Well   - Moderately   - Poorly   - Very poorly   - Don’t know - Ensuring the availability of generic and biosimilar medicines.   - Very well   - Well   - Moderately   - Poorly   - Very poorly   - Don’t know - Ensuring that new medicines are timely available to patients in all EU countries.   - Very well   - Well   - Moderately   - Poorly   - Very poorly   - Don’t know - Ensuring that medicines stay on the market at all times and that there are no shortages.   - Very well   - Well   - Moderately   - Poorly   - Very poorly   - Don’t know - Ensuring that authorised medicines are manufactured, used and disposed of in an environmentally friendly manner.   - Very well   - Well   - Moderately   - Poorly   - Very poorly   - Don’t know - Ensuring that the EU system for development, authorisation and monitoring of medicines, including its rules and procedures, is understandable and easy to navigate.   - Very well   - Well   - Moderately   - Poorly   - Very poorly   - Don’t know - Attracting global investment for medicine innovation in the EU.   - Very well   - Well   - Moderately   - Poorly   - Very poorly   - Don’t know - Is there any other aspect you would like to mention, including positive or unintended effects of the legislation, or would you like to justify your replies?   [Open answer field] |
| **Looking forward** |
| This section reflects on possible solutions to address the problems identified in the inception impact assessment mentioned in the previous section.  Your contribution will help us in defining the way forward.  ________________________________________________  **UNMET MEDICAL NEEDS**  One of the aims of the strategy is to stimulate innovation and breakthrough therapies, especially in areas of ‘unmet medical need’.  Regulators, health technology assessment experts and representatives of bodies responsible for reimbursing or paying for medicines (‘payers’) are discussing a definition or a set of principles for ‘unmet medical needs’ in order to achieve the objectives of the general pharmaceutical legislation. The discussions reveal different perceptions of what is an ‘unmet medical need’. Convergence on this key concept should facilitate the design of clinical trials, generation of evidence and its assessment, and the quick availability on the market of these products and ensuring that innovation matches the needs of patients and of the national health systems.  The purpose of this question is to identify elements that are important in defining what unmet medical need is and in which areas of unmet medical need innovation should be stimulated.  **Q3: How important are the following elements for defining ‘unmet medical needs’?**   - Seriousness of a disease.   - Very important   - Important   - Fairly important   - Slightly important   - Not important   - Don’t know - Absence of satisfactory treatment authorised in the EU.   - Very important   - Important   - Fairly important   - Slightly important   - Not important   - Don’t know - A new medicine has major therapeutic advantage over existing treatment(s).   - Very important   - Important   - Fairly important   - Slightly important   - Not important   - Don’t know - Lack of access for patients across the EU to an authorised treatment.   - Very important   - Important   - Fairly important   - Slightly important   - Not important   - Don’t know - Other (please specify).   - Very important   - Important   - Fairly important   - Slightly important   - Not important   - Don’t know - Is there any other aspect you would like to mention, for example on the potential economic, social, environmental or other impacts of the outlined elements, or would you like to justify your replies?   [Open answer field] |
| **INCENTIVES FOR INNOVATION**  The general pharmaceutical legislation guarantees the pharmaceutical innovator, typically a company, regulatory data and market protection for its new medicinal product. This data protection makes sure that another pharmaceutical company cannot re-use the proprietary data of the innovator for 8 years. Market protection makes sure that a generic or biosimilar medicine cannot be marketed until 10 years after authorisation. This dual protection shields a pharmaceutical innovator from generics or biosimilars on the market for 10 years. This protection is part of the EU system of incentives for innovation. The EU regime of intellectual property protection provides an additional protection coverage but is beyond the scope of this questionnaire and the revision of the general pharmaceutical legislation.  **Q4: What do you think of the following measures to support innovation, including for ‘unmet medical needs’?**   - The current data and market protection periods for innovative medicines: 10 years of market protection, and 8 years of data protection.   - Very important   - Important   - Fairly important   - Slightly important   - Not important   - Don’t know - Provide different data and market protection periods depending on the purpose of the medicine (i.e. longer period of protection in areas of unmet medical need).   - Very important   - Important   - Fairly important   - Slightly important   - Not important   - Don’t know - Reduce the data and market protection periods to allow earlier access for generic and biosimilar medicines to the market.   - Very important   - Important   - Fairly important   - Slightly important   - Not important   - Don’t know - Introduce new types of incentives (e.g., transferable exclusivity vouchers or priority review vouchers) on top of the existing data and market protection for medicines addressing an ‘unmet medical need’.   - Very important   - Important   - Fairly important   - Slightly important   - Not important   - Don’t know - Early scientific support and faster review/authorisation of a new promising medicine for an unmet medical need.   - Very important   - Important   - Fairly important   - Slightly important   - Not important   - Don’t know - Public listing of priority therapeutic areas of high unmet medical need to support product development by providing incentives.   - Very important   - Important   - Fairly important   - Slightly important   - Not important   - Don’t know - Require transparent reporting from companies about their research and development costs and public funding as a condition to obtain certain incentives.   - Very important   - Important   - Fairly important   - Slightly important   - Not important   - Don’t know - Other (please specify)   - Very important   - Important   - Fairly important   - Slightly important   - Not important   - Don’t know - Is there any other aspect you would like to mention, for example on the potential economic, social, environmental or other impacts of the outlined elements, or would you like to justify your replies?   [Open answer field] |
| **ANTIMICROBIAL RESISTANCE**  Antimicrobial resistance (AMR) is the ability of microorganisms (such as bacteria, viruses, fungi or parasites) to survive and grow over time and no longer respond to medicines making infections harder to treat and increasing the risk of infections, severe illness and death. Antimicrobials include antibiotics, which are substances that fight bacterial infections. Overprescribing, overuse and inappropriate use of antibiotics are key drivers of AMR, leading to harmful health outcomes. The question below is intended to collect opinions on both the incentives for the development of new antimicrobials as well as possible option on their prudent use.  **Q5: Should there be specific regulatory incentives for the development of new antimicrobials while taking into account the need for more prudent use and if so what should they be?**  [Open answer field] |
| **FUTURE PROOFING: ADAPTED, AGILE AND PREDICTABLE REGULATORY FRAMEWORK FOR NOVEL PRODUCTS**  Novel products and innovative solutions continue to challenge the understanding of a “medicinal product” with low volume, and cutting-edge products (e.g. medicines combined with self-learning artificial intelligence) becoming a new reality. ‘Bedside’ manufacture of more individualised medicines changes the way medicines are produced. There are classification and interplay challenges with other medical products, such as medical devices and substances of human origin, or related to the combination of clinical trials with in vitro diagnostics/medical devices and medicines. In addition, certain cell-based advanced therapy  Medicines are offered in hospital settings and are exempted from aspects of the pharmaceutical legislation. These developments offer possibilities for novel promising treatments and new ways of authorising and monitoring medicines but they are also testing the limits of the current regulatory system. They need to be addressed to unfold their potential while safeguarding the principles of high quality, safety and efficacy of medicines.  Digital transformation is affecting the discovery, development, manufacture, evidence generation, assessment, supply and use of medicines. Medicines, medical technologies and digital health are becoming increasingly integral to overarching therapeutic options. These include systems based on artificial intelligence for prevention, diagnosis, better treatment, therapeutic monitoring and data for personalised medicines and other healthcare applications.  **Q6: How would you assess the following measures to create an adapted, agile and predictable regulatory framework for novel products?**   - Maintain the current rules.   - Very important   - Important   - Fairly important   - Slightly important   - Not important   - Don’t know - Create a central mechanism in close coordination with other concerned authorities (e.g. those responsible for medical devices, substances of human origins) to provide non-binding scientific advice on whether a treatment/product should be classified as a medicine or not.   - Very important   - Important   - Fairly important   - Slightly important   - Not important   - Don’t know - Make use of the possibility for ‘regulatory sandboxes’  in legislation to pilot certain categories of novel products/technologies.   - Very important   - Important   - Fairly important   - Slightly important   - Not important   - Don’t know - Create adaptive regulatory frameworks (e.g. adapted requirements for authorisation and monitoring with possibility to adjust easily to scientific progress) for certain novel types of medicines or low volume products (hospital preparations) in coherence with other legal frameworks (e.g. medical devices and substances of human origin ) and respecting the principles of quality, safety and efficacy.   - Very important   - Important   - Fairly important   - Slightly important   - Not important   - Don’t know - Introduce an EU-wide centrally coordinated process for early dialogue and more coordination among clinical trial, marketing authorisation, health technology assessment bodies, pricing and reimbursement authorities and payers for integrated medicines development and post-authorisation monitoring.   - Very important   - Important   - Fairly important   - Slightly important   - Not important   - Don’t know - Other (please specify)   - Very important   - Important   - Fairly important   - Slightly important   - Not important   - Don’t know - Is there any other aspect you would like to mention, for example on the potential economic, social, environmental or other impacts of the outlined elements, or would you like to justify your replies?   [Open answer field]  ___________________________________  **Q7: Do you think that certain definitions and the scope of the legislation need to be updated to reflect scientific and technological developments in the sector (e.g. personalised medicines, bedside manufacturing, artificial intelligence) and if so what would you propose to change?**  [Open answer field] |
| **REWARDS AND OBLIGATIONS RELATED TO IMPROVED ACCESS TO MEDICINES**  Some medicines and therapies do not always reach patients in all EU countries, so patients in the EU still have different levels of access to medicines, depending on where they live. Even if a medicine received an EU-wide authorisation, companies are currently not obliged to market it in all EU countries. A company may decide not to market its medicines in, or decide to withdraw them from, one or more countries. This can be due to various factors, such as national pricing and reimbursement policies, size of the population and level of wealth, the organisation of health systems and national administrative procedures. Smaller markets in particular face challenges for availability and supplies of medicines.  **Q8: How would you assess the following measures to improve patient access to medicines across the EU?**   - Maintain the current rules which provide no obligation to market medicines in all EU countries.   - Very important   - Important   - Fairly important   - Slightly important   - Not important   - Don’t know - Require companies to notify their market launch intentions to regulators at the time of the authorisation of the medicine.   - Very important   - Important   - Fairly important   - Slightly important   - Not important   - Don’t know - Introduce incentives for swift market launch across the EU.   - Very important   - Important   - Fairly important   - Slightly important   - Not important   - Don’t know - Allow early introduction of generics in case of delayed market launch of medicines across the EU, while respecting intellectual property rights.   - Very important   - Important   - Fairly important   - Slightly important   - Not important   - Don’t know - Require companies to place – within a certain period after authorisation – a medicine on the market of the majority of Member States, that includes small markets.   - Very important   - Important   - Fairly important   - Slightly important   - Not important   - Don’t know - Require companies withdrawing a medicine from the market to offer another company to taker over the medicine.   - Very important   - Important   - Fairly important   - Slightly important   - Not important   - Don’t know - Introduce rules on electronic product information to replace the paper package leaflet.   - Very important   - Important   - Fairly important   - Slightly important   - Not important   - Don’t know - Introduce harmonised rules for multi-country packages of medicines.   - Very important   - Important   - Fairly important   - Slightly important   - Not important   - Don’t know - Other (please specify).   - Very important   - Important   - Fairly important   - Slightly important   - Not important   - Don’t know - Is there any other aspect you would like to mention, for example on the potential economic, social, environmental or other impacts of the outlined elements, or would you like to justify your replies?   [Open answer field] |
| **ENHANCE THE COMPETITIVE FUNCTIONING OF THE MARKET TO ENSURE AFFORDABLE MEDICINES**  The affordability of medicines has implications for both public and household finances. It poses a growing challenge to pay for medicines in the majority of Member States. Often, innovative medicines have higher prices, while there are growing concerns among stakeholders about the real-life effectiveness of some medicines and related overall costs. This puts the budgetary sustainability of health systems at risk, and reduces the possibilities for patients to have access to these medicines. Generics and biosimilars  of 11 medicines which no longer benefit from intellectual property protection (off-patent medicines) may provide accessible and affordable treatments. They also increase the availability of alternative treatment options for patients. They may also increase competition between available medicines. However, experience shows that there are still barriers for medicines entering the EU market, including for generics or biosimilars.  **Q9: In your view, to what extent would the following measures support access to affordable medicines?**   - Maintain the current rules.   - To a great extent   - To a certain extent   - No change   - Very little   - Not at all   - Don’t know - Stimulate earlier market entry through a broader possibility to authorise generics /biosimilars despite ongoing patent protection (‘Bolar exemption’).   - To a great extent   - To a certain extent   - No change   - Very little   - Not at all   - Don’t know - Create a specific (regulatory) incentive for a limited number of biosimilars that come to the market first.   - To a great extent   - To a certain extent   - No change   - Very little   - Not at all   - Don’t know - Introduce an EU-wide scientific recommendation on interchangeability for specific biosimilars.   - To a great extent   - To a certain extent   - No change   - Very little   - Not at all   - Don’t know - Introduce other, non-legislative measures, such as joint procurement to reinforce competition while addressing security of supply and environmental challenges.   - To a great extent   - To a certain extent   - No change   - Very little   - Not at all   - Don’t know - Other (please specify).   - To a great extent   - To a certain extent   - No change   - Very little   - Not at all   - Don’t know - Is there any other aspect you would like to mention, for example on the potential economic, social, environmental or other impacts of the outlined elements, or would you like to justify your replies?   [Open answer field] |
| **REPURPOSING OF MEDICINES**  Repurposing is the process of identifying a new use for an established medicine in a disease or condition other than that it is currently authorised for. Repurposing of older (off-patent) medicines constitutes an emerging and dynamic field of medicines development, often led by academic units and medical research charities, with the potential for faster development times and reduced costs as well as lower risks for companies. This is because repurposing commonly starts with substances that have already been tested and many have demonstrated an acceptable level of safety and tolerability. The objective is to identify the opportunities and address any regulatory burdens to facilitate repurposing of off-patent, affordable medicines.  **Q10: What measures could stimulate the repurposing of off-patent medicines and provide additional uses of the medicine against new diseases and medical conditions? Please justify your answers.**  [Open answer field] |
| **SECURITY OF SUPPLY OF MEDICINES**  Shortages of medicines and the vulnerabilities in the pharmaceutical supply chain continue to be concerns in the EU. Shortages of medicines can have serious impacts on patient care. Under the current pharmaceutical legislation, pharmaceutical companies and wholesalers must, within the limits of their responsibilities, ensure a continued supply of medicines once they are placed on the market in the EU. Companies must also notify national authorities at least two months before an expected shortage or planned market withdrawal.  **Q11: What is your view on the following measures to ensure security of supply of medicines in the EU?**   - Maintain the current rules.   - Very important   - Important   - Fairly important   - Slightly important   - Not important   - Don’t know - Earlier reporting of shortages and market withdrawals to national authorities in a common format.   - Very important   - Important   - Fairly important   - Slightly important   - Not important   - Don’t know - Companies to have shortage prevention plans.   - Very important   - Important   - Fairly important   - Slightly important   - Not important   - Don’t know - Companies to have safety stocks.   - Very important   - Important   - Fairly important   - Slightly important   - Not important   - Don’t know - Monitoring of supply and demand at national level.   - Very important   - Important   - Fairly important   - Slightly important   - Not important   - Don’t know - Introduce a shortage monitoring system at EU level.   - Very important   - Important   - Fairly important   - Slightly important   - Not important   - Don’t know - Require companies to diversify their supply chains, in particular the number of key suppliers of medicines and components.   - Very important   - Important   - Fairly important   - Slightly important   - Not important   - Don’t know - Companies to provide more information to regulators on their supply chain.   - Very important   - Important   - Fairly important   - Slightly important   - Not important   - Don’t know - Introduce penalties for non-compliance by companies with proposed new obligations.   - Very important   - Important   - Fairly important   - Slightly important   - Not important   - Don’t know - EU coordination to help identify areas where consolidation in the supply chain has reduced the number of suppliers.   - Very important   - Important   - Fairly important   - Slightly important   - Not important   - Don’t know - Other (please specify).   - Very important   - Important   - Fairly important   - Slightly important   - Not important   - Don’t know - Is there any other aspect you would like to mention, for example on the potential economic, social, environmental or other impacts of the outlined elements, or would you like to justify your replies?   [Open answer field] |
| **QUALITY AND MANUFACTURING**  Medicines manufactured for the EU market must comply with the principles and guidelines of good manufacturing practice (GMP). GMP describes the minimum standard that a medicines manufacturer must meet in their production processes. GMP requires that medicines are of consistent high quality, are appropriate for their intended use and meet the requirements of the marketing authorisation or clinical trial authorisation.  **Q12: What is your opinion of the following measures to ensure manufacturing and distribution of high quality products?**   - Maintain the current rules.   - Very adequate   - Adequate   - Neutral   - Less adequate   - Not adesuate   - Don’t know - Strengthen manufacturing and oversight rules.   - Very adequate   - Adequate   - Neutral   - Less adequate   - Not adesuate   - Don’t know - Adapt manufacturing rules to reflect new manufacturing methods.   - Very adequate   - Adequate   - Neutral   - Less adequate   - Not adesuate   - Don’t know - Include selected environmental requirements for manufacturing of medicines in line with the one health approach on antimicrobial resistance.   - Very adequate   - Adequate   - Neutral   - Less adequate   - Not adesuate   - Don’t know - Increase Member State cooperation and surveillance of the supply chain in the EU and third countries.   - Very adequate   - Adequate   - Neutral   - Less adequate   - Not adesuate   - Don’t know - Strengthen and clarify responsibilities of business operators over the entire supply chain on sharing information on quality, safety and efficacy.   - Very adequate   - Adequate   - Neutral   - Less adequate   - Not adesuate   - Don’t know - Other (please specify).   - Very adequate   - Adequate   - Neutral   - Less adequate   - Not adesuate   - Don’t know - Is there any other aspect you would like to mention, for example on the potential economic, social, environmental or other impacts of the outlined elements, or would you like to justify your replies?   [Open answer field] |
| **ENVIRONMENTAL CHALLENGES**  While access to pharmaceuticals is a priority, it is also important that the environmental impacts of those pharmaceuticals are as low as possible. The environmental risk assessments (ERAs) is currently not taken into account in the overall benefit/risk analysis which influences the delivery of a marketing authorisation (MA) of a medicine. ERA can influence risk management measures. Yet, ERA results are not decisive in the MA process.  **Q13: How would you assess the following measures to ensure that the environmental challenges emerging from human medicines are addressed?**   - Maintain the current rules.   - Very important   - Important   - Fairly important   - Slightly important   - Not important   - Don’t know - Strengthen the environmental risk assessment during authorisation of a medicine, including risk mitigation measures, where appropriate.   - Very important   - Important   - Fairly important   - Slightly important   - Not important   - Don’t know - Harmonize environmental risk assessment by national regulators, including risk mitigation measures.   - Very important   - Important   - Fairly important   - Slightly important   - Not important   - Don’t know - Increase information to the health care professionals and the general public about the assessment of environmental risks of medicines.   - Very important   - Important   - Fairly important   - Slightly important   - Not important   - Don’t know - Allow companies to use existing data about environmental risks for authorisations of a new medicine to avoid duplicating tests.   - Very important   - Important   - Fairly important   - Slightly important   - Not important   - Don’t know - Other (please specify).   - Very important   - Important   - Fairly important   - Slightly important   - Not important   - Don’t know - Is there any other aspect you would like to mention, for example on the potential economic, social, environmental or other impacts of the outlined elements, or would you like to justify your replies?   [Open answer field] |
| **Q14: Is there anything else you would like to add that has not been covered in this consultation?**  [Open answer field] |
| **Q15: In case you would like to share a document that substantiates your replies, please upload it below (optional).**  [Open answer field] |
